# A new paradigm for marine ecological monitoring through swarm intelligence, digital twins, and Human–Swarm interaction

**DOI:** 10.3389/frobt.2026.1809951

**Published:** 2026-06-30

**Authors:** Micael S. Couceiro, Faisal Mazloum, André G. Araújo, Rui P. Rocha, Ana Bio, Gonçalo Ferreira

**Affiliations:** 1 Ingeniarius, Ltd., Alfena, Portugal; 2 Department of Electrical & Computer Engineering, Institute of Systems and Robotics (ISR), University of Coimbra, Coimbra, Portugal; 3 Interdisciplinary Centre of Marine and Environmental Research (CIIMAR), University of Porto, Matosinhos, Portugal; 4 Riamaris, Ltd., Matosinhos, Portugal

**Keywords:** autonomous surface vehicles, digital twins, environmental sensing, human-swarm interaction, marine ecological monitoring, swarm robotics

## Abstract

Marine and coastal ecosystems are among the least observable yet most rapidly changing environments, where climate impacts, pollution, and biodiversity loss demand monitoring and intervention at scales that manual sampling and single-robot deployments cannot sustain. This paper argues for a conceptual shift in ecological monitoring and restoration toward networked robotic ecosystems, adopting cooperative swarms of autonomous aquatic robots coupled to *in-situ* digital twins and human-in-the-loop supervision. We use the REMORA project as a concrete instantiation of this paradigm, outlining an integrated architecture in which multiple low-cost, persistent robots perform distributed sensing and targeted interventions, while a continuously updated digital twin fuses multi-source data to support predictive assessment, “what-if” scenario exploration, and decision support. A dedicated human-swarm interaction layer enables non-roboticist stakeholders (e.g., environmental managers and aquaculture operators) to specify intent, manage exceptions, and maintain trust without micromanaging individual units. By linking advances in swarm intelligence, sensor integration, AI-enabled analysis, and interactive decision-making, the proposed approach targets durable, scalable, and socially relevant solutions for ecological monitoring and ecosystem management, spanning aquaculture, marinas, and broader coastal environments, and provides a roadmap for translating these technologies from controlled trials to sustained field operations.

## 1 Introduction

Marine ecosystems are under increasing pressure from human activity and climate change, necessitating more advanced tools for monitoring and management Crain et al. (2009). Traditional marine robotics approaches (e.g., a single autonomous vehicle or fixed sensor networks) cannot alone provide the required spatiotemporal monitoring resolution or adaptability at reasonable cost. Fixed sensors and single robots, while useful, have inherent limitations, static sensing cannot track dynamic events and both cannot achieve high spatial and temporal data resolution across large areas Duarte et al. (2016). This highlights the need for a new approach to observing and safeguarding marine environments.

We argue that a paradigm shift grounded in swarm intelligence, digital twins, and human-in-the-loop control is needed to meet these challenges.

- Swarm intelligence: Deploying large-scale fleets of inexpensive, autonomous marine robots offers scalability, fault-tolerance, and wide-area coverage beyond the capabilities of individual vehicles. By relying on decentralised control and local communication, swarms can adapt to dynamic ocean conditions in real time and effectively cover vast areas Duarte et al. (2016).
- Digital twins: A digital twin (DT) is essentially a virtual counterpart of a physical asset or system that fuses real-time sensor data with computational models, providing enhanced insight into system dynamics and predictive analytics for decision support. Integrating DT technology into marine robotics enables operators to simulate “what-if” scenarios, optimise strategies, and anticipate changes in the marine environment Føre et al. (2024).
- Human-in-the-loop control: Equally important is leveraging human expertise and oversight within the loop. Effective human-swarm interaction (HSI) ensures that autonomous behaviours align with mission objectives and ethical considerations. The human element is often essential for guiding swarm operations in complex, unstructured environments, combining human intuition with robotic efficiency for superior outcomes Hussein and Abbass (2018).

As a Perspective paper, this manuscript does not claim a completed experimental validation of REMORA, but rather presents a systems-level vision, architecture, and validation roadmap for translating swarm robotics, DTs, and HSI into marine ecological monitoring. The contribution of REMORA is not the introduction of a new standalone swarm controller or a new standalone digital-twin estimator, but rather a framework in which these components are conceived, integrated, and operated as a single closed loop. Specifically, REMORA advances an architecture that couples: (i) heterogeneous aquatic robot swarms for persistent, distributed sensing and targeted intervention; (ii) an *in-situ* DT designed as an operational component for macroscopic state estimation, spatio-temporal data fusion, and predictive “what-if” analysis; and (iii) a HSI layer through which non-roboticist stakeholders can supervise missions, bias collective behaviour, and act on DT-informed insights without micromanaging individual robots. In this sense, the novelty of REMORA is primarily architectural and integrative: it frames swarm autonomy, environmental digital modelling, and operator oversight not as parallel research threads, but as mutually dependent layers of a deployable monitoring ecosystem. This perspective aligns directly with the Research Topic *Robotic Advancements in Ecological Monitoring and Ecosystem Restoration*, by proposing a durable, scalable, and socially grounded pathway for translating robotic and AI capabilities into ecologically meaningful practice.

## 2 Marine robotics

Marine robotic systems, generally encompassing Autonomous Underwater Vehicles (AUVs) and Autonomous Surface Vehicles (ASVs), have greatly expanded our capacity for ocean observation and exploration. These robots are now routinely used for tasks like seabed mapping, water-quality monitoring, and infrastructure inspection in environments ranging from coastal waters to deep-sea and under-ice regions. However, most deployments still involve a single vehicle tackling one mission at a time, often with low autonomy and direct human supervision (or manual remote control). As a result, achieving persistent and large-scale environmental monitoring with traditional marine robotics remains challenging. Recent trends point toward a shift from single-vehicle autonomy to multi-vehicle cooperation, aiming to increase the area covered, the frequency of data collection, and the resilience of operations.

### 2.1 Challenges

Current marine robotic approaches for environmental monitoring face several key challenges:

- Labour-Intensive, Low-Data Operations: Traditional monitoring often relies on manual sampling or one-robot-at-a-time surveys, which are costly and time consuming. For example, regular water sampling campaigns require boats, crew, and favourable weather, yet yield only sparse data in space and time Morales-Aragón et al. (2024). Conventional methods, like diver-based and single-UAV surveys, though labour-intensive, simply cannot provide the necessary scale or frequency of data, missing many changes between samplings Sultan et al. (2025).
- Insufficient Spatiotemporal Coverage: Because sampling is infrequent and localised, fast or subtle ecosystem dynamics cannot be captured and important environmental events can go undetected. In one case, weekly manual profiling of a coastal lagoon produced such a low sampling frequency that many transient phenomena were never observed, undermining ecosystem assessment Morales-Aragón et al. (2024). Continuous high-frequency monitoring is rarely feasible with these traditional methods, creating blind spots in our understanding of the environment. Even fixed sensor networks, while operating continuously, are installed at discrete locations and cannot reconfigure or move to track dynamic events Duarte et al. (2016). A single AUV or static sensor array thus offers only a fragmentary view of a complex, dynamic marine ecosystem.
- Limited Scalability and Resilience: Operating one vehicle at a time also means there is no built-in redundancy. If that sole robot malfunctions or if conditions prevent its deployment, data collection halts entirely. With no overlapping coverage or backup units, the system lacks robustness against failures or harsh conditions. Achieving persistent monitoring would require multiple platforms working in parallel, which current one-at-a-time approaches do not support. These shortcomings have prompted calls for automated, scalable monitoring methods that can increase the volume of data collected while reducing the reliance on human effort Zereik et al. (2018).

Collectively, the above challenges highlight that traditional marine robotic operations struggle to provide continuous, wide-ranging, and reliable environmental data. Beyond these first-order limitations, the transition toward persistent multi-robot ecological monitoring introduces a second layer of tightly coupled challenges. First, even when focusing on surface systems, communication remains fragile under dynamic MANET conditions, where topology changes, partial occlusions, link degradation, and intermittent multi-hop connectivity can directly affect coordination and data consistency. Second, scalability is constrained not only by robot count, but also by endurance and maintainability: increasing swarm size improves coverage and redundancy, but also amplifies power-management, solar-harvesting variability, payload/energy trade-offs, and degradation phenomena such as bio-fouling. Third, the DT layer is only as reliable as its synchronisation and input quality; asynchronous sensing, sensor drift, missing data, and model mismatch can propagate uncertainty into state estimation and prediction, which is particularly critical in ecological monitoring where weak or slowly evolving signals may still be operationally relevant. Fourth, HSI introduces its own bottleneck, since the operator must supervise not only multiple robots, but also swarm state, environmental indicators, and DT-generated alerts, making cognitive load, trust, and timely intervention central design constraints rather than secondary usability concerns. These open issues reinforce the need for architectures in which communications, autonomy, digital modelling, and supervision are co-designed as a single operational ecosystem rather than treated as loosely connected add-ons.

### 2.2 The need for a new paradigm

Swarm robotics offers a fundamentally different approach with inherent advantages for marine monitoring. In a swarm, many inexpensive robots cooperate autonomously, inspired by the collective behaviours and emergent cooperation of natural swarms, like insect swarms, fish shoals or bird flocks Ramesh et al. (2025). Instead of a single complex robot, a swarm uses numerous simple robotic units, i.e., robotic agents, that coordinate through local interactions and decentralised control. This design brings several benefits, such as (i) scalability, because the same simple rules can govern a few or a few hundred robots; (ii) adaptability, as the group can self-organise and cooperatively respond to environmental changes; and (iii) robustness, since the failure of any individual has little effect on the overall mission. For instance, a swarm of aquatic robots can efficiently spread out to cover a large area, collecting data simultaneously at multiple locations, which dramatically increases the spatial and temporal resolution of observations beyond what a single vehicle could achieve Duarte et al. (2016). The redundancy of having many units also provides fault tolerance, as even if some robots are lost or incapacitated, the remainder can continue the mission with minimal performance loss. In short, a cooperative multi-robot system can gather far more data with higher frequency and resilience than isolated deployments.

Despite these promising attributes, the real-world deployment of marine robot swarms has been slow. To date, most swarm robotics research has been confined to laboratories and simulations, with relatively few examples of large robot swarms operating in the wild Sànchez et al. (2018). One of the primary hurdles has been the lack of effective HSI mechanisms for supervision and control. Orchestrating dozens or hundreds of autonomous robots poses significant challenges for operators, from tracking and being aware of the macroscopic state of the swarm to intervening when necessary, and traditional human–robot interfaces are not equipped for this scale Kolling et al. (2015). Without intuitive tools for a human to supervise and control a swarm, field adoption has remained limited. This indicates that technical progress alone (in robot hardware or swarm algorithms) is not enough; new paradigms for HSI are needed to unlock the full potential of robotic swarms in marine environments and other real-world applications.

In light of these considerations, we argue for a paradigm shift in marine robotics: moving from isolated, single-purpose robots toward interconnected swarms of marine robots tightly integrated with advanced digital modelling and operator oversight. By linking a swarm to digital models (e.g., real-time environmental maps or predictive simulations), an operator can gain a high-level situational awareness of both the robots and their environment, making it feasible to manage complex multi-robot missions. The swarm intelligence principles described above, such as adaptive flocking (maintaining cohesive group motion) and dispersion (spreading out to maximise coverage), provide the foundation for this new approach. In practice, this means designing marine robot collectives that can autonomously coordinate their movements and implement distributed adaptive sampling strategies, while allowing humans to interact with the swarm as a whole (rather than micromanaging individuals). This human-swarm partnership would enable continuous, high-resolution monitoring of marine ecosystems at scales unachievable by traditional means, effectively addressing the challenges outlined earlier.

## 3 The REMORA vision

**FIGURE 1:**
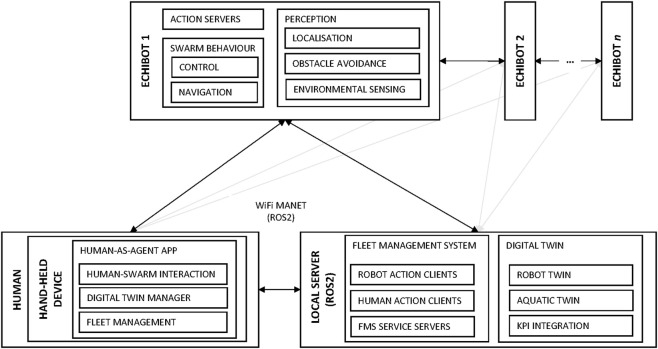
Reference architecture of the REMORA system. At the microscopic level, multiple EchiBots execute local perception, navigation, obstacle avoidance, environmental sensing, and swarm behaviours while exchanging information through a WiFi MANET built on ROS2. At the macroscopic level, a Local Server hosts the FMS and the DT (Aquatic Twin, Robot Twin, and KPI Integration), whereas a human hand-held device runs a Human-as-Agent App that integrates HSI, fleet management interaction, and digital-twin supervision to close the mission-level feedback loop.

REMORA introduces a distributed cyber-physical architecture that couples autonomous swarm robotics, an *in-situ* digital twin (DT), and human–swarm interaction (HSI) into a single operational loop for aquatic ecosystem monitoring and intervention. As shown in Figure 1, the architecture establishes three coupled feedback structures. First, each EchiBot closes a *local autonomy loop*, in which perception, localisation, obstacle avoidance, and environmental sensing support the execution of action servers and swarm-level control laws. Second, a *macroscopic coordination loop* is realised through the fleet management system (FMS) and the DT, where robot telemetry and environmental observations are aggregated, synchronised, and transformed into a global representation of the swarm and the aquatic system. Third, a *human supervisory loop* is closed through the Human-as-Agent App, which allows the operator to visualise mission and DT state, inspect key performance indicators (KPIs) and alerts, and inject high-level commands or parameter changes back into the system. In practical terms, this means that the main outputs of the swarm layer are robot state, environmental observations, and local connectivity information, whereas the main inputs injected from the supervisory side are mission goals, behaviour selection, and reconfiguration commands.

At the current project stage, implemented or initiated components include the first EchiBot prototype, the Unity-ROS2 simulation/DT baseline, and early ROS2swarm integration, whereas planned developments include full multi-robot field deployment, data-assimilative DT operation, and end-user-centred HSI validation.

### 3.1 Swarm-intelligent robotic platform

**FIGURE 2:**
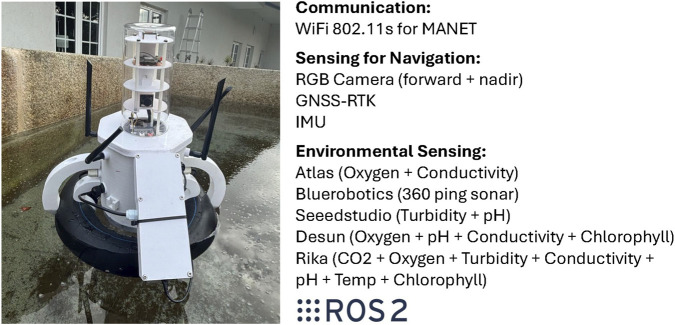
EchiBot: REMORA’s ROS2-based aquatic swarm unit with a reconfigurable sensing and communications stack for long-duration deployments in confined and semi-natural waters.

At the core of REMORA is a swarm of autonomous aquatic robots equipped with sensors and actuators for water quality monitoring and fish shoal management. A first prototype, *EchiBot*, has been iteratively developed and tested on an aquatic 3×3 m testbed (Figure 2). Each unit is conceived as a modular ROS2-enabled platform with two complementary sensing layers: (i) navigation and situational awareness, combining RTK-capable GNSS, IMU, and forward/nadir RGB cameras; and (ii) multi-parametric environmental sensing, configurable with commercial probes for dissolved oxygen and conductivity (e.g., Atlas), turbidity and pH (e.g., Seeedstudio), and extended packages including chlorophyll, temperature, and ${\text{CO}}_{2}$ (e.g., Rika). A 360 ° acoustic ranging sensor (BlueRobotics Ping sonar) complements these modalities for near-field obstacle/structure awareness and water-column interrogation.

Architecturally, each EchiBot is not treated merely as a sensing buoy, but as a self-contained ROS2 node in the swarm, decomposed into three functional blocks that are also made explicit in Figure 1: *Perception*, *Action Servers*, and *Swarm Behaviour*. Perception integrates localisation, obstacle awareness, and environmental sensing into a local estimate of the robot and its surroundings; Action Servers expose long-running robot capabilities such as navigation or mission execution through ROS2-compatible interfaces; and Swarm Behaviour implements the local interaction rules that generate collective phenomena such as flocking, coverage, aggregation, and connectivity-preserving motion. This decomposition is important because it separates robot-level reactivity from swarm-level coordination and enables the FMS to interact with the robots through standardised ROS2 topics, services, and actions rather than through direct teleoperation.

To coordinate as a group, the robots communicate over a Mobile Ad Hoc Network (MANET) providing distributed, infrastructure-free connectivity under dynamic topology Boukerche et al. (2011). We adopt IEEE 802.11s in mesh mode and B.A.T.M.A.N. advanced (batman-adv) for decentralised Layer two routing Seither et al. (2011), exposing the swarm as a common L2 domain despite multi-hop forwarding. This simplifies ROS2/DDS integration and supports automatic next-hop selection and fast re-routing as links vary, with each robot acting as both end node and relay. Beyond connectivity, the MANET is treated as a first-class subsystem: robots estimate neighbourhood connectivity (presence/link quality) and couple it with local sensing to sustain swarm behaviours under intermittent communications, including network-aware policies that preserve graph connectivity (e.g., “stay connected”) during coverage, adaptive sampling, and event-driven re-tasking.

REMORA leverages open-source frameworks, such as ROS2swarm Kaiser et al. (2022), to implement real-time distributed control. During the first 6 months, ROS2swarm was refined to improve robustness at the hardware-protection layer by: (i) correcting repulsive-vector computation/normalisation to avoid angular-rate saturation; (ii) resolving quadrant ambiguities in the asin()-based heading logic; and (iii) separating robot-robot from robot-environment range interfaces to reduce interference between swarm motion primitives and reactive avoidance.

### 3.2 Digital twin integration

A key innovation of REMORA is the development of an *in-situ* DT that jointly represents (i) the aquatic system and (ii) the swarm itself, being designed from the outset as an operational component rather than a *post hoc* visualisation tool. Architecturally, REMORA structures the DT stack into three coupled layers:

- DT parametrisation: where the relevant state variables (e.g., dissolved oxygen, temperature, salinity, chlorophyll-A, nutrients) and the required temporal/spatial resolutions are defined and harmonised across pilots.
- DT of the aquatic system: where multi-source measurements from the robots (and external probes where available) are assimilated to estimate the spatio-temporal evolution of the environment, fish distribution/behaviour and morphology.
- DT of the robot and integrator DT: where robot kinematic/dynamic models (including non-linearities) and sensor models (including error characterisation) are integrated and exposed through standardised interfaces and data protocols to support real-time 3D visualisation, simulation, and predictive analysis.

This layered view is aligned with REMORA’s plan to transmit, store, and process large sensing volumes efficiently, while enabling AI-based classification and predictive analytics, such as forecasting KPIs related to fish health. Importantly, the DT is conceived here as primarily physics-based: the Aquatic Twin aims to represent the monitored water body at a macroscopic level, while the Robot Twin captures the relevant physical, kinematic, sensorial, and mission-related state of the robotic units. In this sense, the DT is not intended as a purely data-driven replica, but as a physically grounded computational model that can be updated online using field measurements.

In the reference architecture (Figure 1), the DT is hosted at the Local Server and is explicitly decomposed into three blocks: the *Aquatic Twin*, the *Robot Twin*, and *KPI Integration*. The Aquatic Twin captures the macroscopic state of the monitored environment, including water-quality variables and relevant ecological or infrastructural features; the Robot Twin represents the state, configuration, and expected behaviour of the robotic units; and KPI Integration provides a bridge between raw measurements, derived indicators, and operator-facing mission assessment. This decomposition is technically relevant because it makes the DT more than a visual replica: it becomes the server-side mechanism through which robot telemetry, environmental observations, and mission state are synchronised into a common representation that can support scenario evaluation, anomaly tracking, and decision support.

To keep the DT aligned with field conditions, REMORA follows a data-assimilation viewpoint. Let $E_{t}^{f}$ denote the forecast (or prior) environmental state propagated by the DT model before assimilating the latest observations, $E_{t}^{a}$ the analysis (or posterior) state obtained after incorporating those observations, $y_{t}$ the set of time-stamped observations provided by the swarm at time t , and $\theta_{t}$ a set of selected model parameters. In abstract form, the DT may be described as a forecast-analysis loop through Equations 1–3:

$$E_{t}^{f}=M\left(E_{t-1}^{a},\theta_{t}\right)+w_{t},$$ (1)

$$y_{t}=H\left(E_{t}^{f}\right)+v_{t},$$ (2)

$$E_{t}^{a}=E_{t}^{f}+K_{t}\left(y_{t}-H\left(E_{t}^{f}\right)\right),$$ (3)

where M denotes the physics-based temporal propagation of the aquatic state, H an observation operator, $K_{t}$ an update gain, and $w_{t},v_{t}$ model and measurement uncertainty terms, respectively. The purpose of this abstraction is not to commit the project, at this stage, to a single filter family, but rather to make explicit that REMORA’s DT is designed as an online-updated estimate rather than an offline post-processing artefact.

A representative instantiation is dissolved oxygen monitoring in an aquaculture pond or marina basin. In that case, $E_{t}$ may be interpreted as a spatial field of dissolved oxygen over the monitored area; M propagates this field using a simplified physics-based transport/dispersion model with source–sink terms; $y_{t}$ contains time-stamped oxygen measurements collected by the EchiBots at their current locations; and H maps the DT field to those sampling locations. Operationally, the DT would maintain not only the estimated field $E_{t}^{a}$ , but also an associated uncertainty or confidence layer. During forecast propagation, or when robots are disconnected and observations are missing, uncertainty increases according to model and process noise; when new measurements are assimilated, uncertainty decreases locally around the observed regions and can be used to guide subsequent adaptive sampling. Thus, uncertainty is not treated only as an offline error statistic, but as an operational variable that can influence where the swarm should sample next.

In the first 6 months of REMORA, the DT effort has been grounded on a realistic Unity-based simulator as an initial DT instantiation for rapid closed-loop development and risk reduction. The current implementation already includes the EchiBot platforms and environmental effects relevant to aquatic operation (e.g., water and wind), and is integrated with ROS2, enabling control of the simulated robots using mechanisms compatible with the forthcoming integration of swarm algorithms and HSI modules. This work directly supports the DT-of-the-robot and integrator-DT tasks, whose execution requires progressive modelling and integration of sensors and behaviours in simulation, while keeping the software interfaces consistent with the physical stack.

At the implementation level, this means that REMORA maintains the DT within the same ROS2-centred integration backbone as the swarm and the operator interface, avoiding fragmentation across heterogeneous middleware. DT synchronisation is therefore not conceived as an external post-processing step, but as part of the operational loop: robot telemetry and environmental data are propagated through the MANET to the Local Server, incorporated into the DT, and then exposed to the FMS and the operator through ROS2-compatible interfaces. Because REMORA adopts a distributed architecture, however, perfect synchronisation cannot be assumed. Network partitions, delayed packets, and asynchronous sensing may temporarily yield incomplete updates of the DT. In such cases, the physics-based model continues to propagate the best available estimate forward in time, while uncertainty is expected to grow under missing observations and to decrease once buffered measurements are re-assimilated.

Within this perspective, REMORA treats uncertainty as a first-class concern rather than an implementation detail. Part of this uncertainty arises from model mismatch and sensor drift; part of it emerges from incomplete coverage accomplished by the robot swarm or delayed observations in the MANET. The swarm itself is expected to help mitigate these effects through redundancy and spatial diversity of measurements: overlapping observations from multiple robots can support cross-validation, basic consistency checks, and quality gating of incoming data before assimilation. This is particularly relevant in ecological monitoring, where weak or slowly evolving environmental signals may still be operationally significant and should not be discarded solely because they are locally sparse or partially delayed.

REMORA will evolve this DT from a simulation-first baseline into a data-assimilative, decision-support digital twin for the two target pilots (further described in Section 3.4). More specifically, ongoing and planned steps include completing DT parametrisation and implementing the aquatic DT with explicit treatment of spatio-temporal resolution and uncertainty. This should be followed by deploying AI modules for classification and predictive analytics within the DT loop, enabling “what-if” scenario evaluation (e.g., feeding strategy adjustments, anomaly propagation) and supporting risk-aware operation through KPI-driven monitoring. In this context, AI is foreseen mainly as a hybrid augmentation of the DT, for example, through surrogate modelling to reduce computational burden, residual/parameter-update models to compensate model mismatch, or anomaly/outlier detection over degraded sensor streams, rather than as a replacement for the physics-based core. Importantly, REMORA treats Unity as both (a) the preferred simulation engine for aquatic-specific physics/visual realism and (b) the front-end substrate for DT-driven UI/UX in real-time supervision, ensuring continuity between development, validation and field operation.

### 3.3 Human-swarm interaction

In the reference architecture (Figure 1), HSI is operationalised through the *Human-as-Agent App*, which groups together three complementary functions: *HSI*, *Fleet Management*, and *DT Manager*. This design choice reflects the fact that the human operator is not expected to interact with individual robots directly, but rather with the swarm as a coordinated system. Concretely, the operator-facing layer is responsible for mission creation and parametrisation, start/stop and reconfiguration commands, selection of collective behaviours, adjusting the swarm behaviour performance during the collective task execution through parameter setting or persistent influence via temporary leaders, visualisation of the swarm and DT state, and inspection of KPIs, alerts, and mission progress. These interactions are mapped onto ROS2 topics, services, and actions, allowing the operator layer to remain tightly coupled to the FMS and DT while preserving the distributed execution of the swarm. In practical terms, the interface is intended to translate stakeholder intent into swarm-level commands - for example, defining where to monitor, which collective behaviour to prioritise, when to investigate an alert, and how strongly to bias the swarm toward a region of interest - while hiding robot-by-robot coordination details from the user.

REMORA adopts remote HSI as the default operational mode, reflecting the target scenarios (marinas and aquaculture tanks) where proximity can be impractical, unsafe, or intrusive. Technically, HSI is designed as a closed-loop architecture coupling (i) local swarm control laws running onboard each robot; (ii) a decentralised mechanism for macroscopic state estimation over the MANET; and (iii) a Unity-based operator UI fed by the integrated DT.

A central element is the development of a decentralised, self-organised data aggregation (DeSODA) mechanism designed to be scalable to very large robot swarms (e.g., hundreds of robots) and grounded on the locality principle: robots acquire variables locally and DeSODA propagate them through short-range, multi-hop exchanges, where selected nodes aggregate neighbourhood information and forward it towards the base station/DT, yielding a macroscopic estimate of swarm and environment state without requiring global knowledge at each robot. In REMORA, the most important swarm behaviour is adaptive data sampling wherein robots cooperate to effectively sample spatiotemporal phenomena in aquatic ecosystems (e.g., dissolved oxygen, salinity, etc.) Manjanna et al. (2022). These data samples are aggregated and relayed by the swarm to the base station through a MANET, thus feeding the DT with the required data. At the same time, as part of the DeSODA mechanism, robots self-organise themselves in small clusters to locally aggregate data in an aggregator robot, which estimates a simplified spatiotemporal model used by the robots within the cluster to take informed, coordinated decisions about where to sample next. This mechanism is explicitly foreseen to interface the DT and the HSI stack, and to minimise operator workload while still enabling supervision at swarm level, with quantitative targets for operator trust Schaefer (2016), operator workload Karakikes and Nathanael (2023), and usability Di Nuovo et al. (2019) guiding design and evaluation.

From a control perspective, REMORA treats HSI as a supervision problem over emergent collective behaviour. Rather than relying on global knowledge at each robot, the architecture foresees a decentralised aggregation mechanism over the MANET, grounded on locality-based exchanges, to provide the operator with a macroscopic estimate of swarm and environment state with manageable workload. This makes it possible for the operator to influence the swarm through higher-level interaction primitives, such as behaviour selection, parameter adjustment, or persistent influence via temporary leaders, instead of micromanaging individual trajectories. In this sense, HSI in REMORA is conceived as the bridge between microscopic local interactions and macroscopic collective behaviour, closing the loop between sensing, digital representation, and mission-level decision making.

In addition to scalability to large robot swarms, a key requirement for the deployment of swarm robotics in large-scale applications, the methods developed within REMORA for HSI aim to support operator situation awareness of both the swarm state and collective task execution status, while enabling macroscopic-level control of swarm behaviour. At the same time, these methods seek to maintain operator workload at practical and acceptable levels. This objective represents a core requirement of the REMORA project and is operationalised through the inclusion of the NASA Task Load Index (NASA-TLX) in the project’s validation framework Hart and Staveland (1988).

From an implementation standpoint, this roadmap is being anchored in the ROS2 ecosystem: early ramp-up work already integrated ROS2swarm with scalable (Stage) and high-fidelity (Gazebo/Unity) simulators and introduced network-aware behaviour patterns to ensure that HSI remains feasible under dynamic multi-hop connectivity provided by a MANET; in parallel, Unity-ROS2 integration is being used as the basis for the operator-facing UI and DT-driven visualisation, ensuring continuity between simulation-first development and later field operation.

### 3.4 Evaluation and validation strategy

Although this paper does not report a completed experimental campaign, the REMORA vision is anchored in a concrete validation pathway intended to make the proposed architecture measurable, comparable, and progressively falsifiable. At the current stage, quantitative pilot-level results, such as single-robot versus swarm coverage comparisons or DT estimation-consistency plots, would be premature: the project is still transitioning from early component development and simulation-first integration toward calibrated multi-robot field operation. Implemented or initiated assets, including the EchiBot prototype, the ROS2-based communications stack, the Unity-connected DT baseline, and preliminary swarm simulations, are already being used to de-risk this transition, but they do not yet provide representative evidence of full-system performance under realistic aquaculture or marina conditions. For this reason, we avoid reporting illustrative metrics that could overstate maturity, and instead define the validation axes, targets, and benchmark logic that will guide subsequent controlled and field evaluations.

Table 1 summarises the core validation axes foreseen for REMORA. Four primary KPIs are used to structure this pathway. First, the swarm robotic platform will be assessed through *Average Path Deviation (APD)*, targeting trajectory-execution performance below 10%, thereby quantifying the fidelity with which coordinated robotic units can execute navigation and monitoring behaviours Pacheco and Luo (2015). Second, the DT will be evaluated through *Normalised Root Mean Square Error (NRMSE)*, with a target below 0.1, comparing DT estimates and predictions against *in-situ* measurements gathered during deployment Munoz (2022). Third, the HSI layer will be assessed through the *Trust Perception Scale for Human-Robot Interaction (TPS-HRI)*, targeting scores above 60%, in order to determine whether operators perceive the system as sufficiently trustworthy for supervision at swarm level Schaefer (2016). Operator workload will also be assessed using the *NASA Task Load Index (NASA-TLX)*, targeting scores below 50/100 to ensure that swarm supervision and interaction remain cognitively and operationally manageable Hart and Staveland (1988). Fourth, the Unity-based interface will be evaluated through the *System Usability Scale (SUS)*, targeting values above 68%, to ensure that the supervisory layer remains usable by non-roboticist stakeholders Di Nuovo et al. (2019). Nevertheless, these instruments are inherently subjective and may be affected by prior robotics experience, task familiarity, novelty effects, and user expectations. Therefore, they will be interpreted together with objective supervisory indicators, including intervention frequency, alert acknowledgement/response time, behaviour-reconfiguration time, and supervised mission completion.

**TABLE 1:** Core validation axes foreseen for REMORA. The first four rows correspond to the primary project KPIs, while the last row lists complementary operational indicators that will be reported to characterise real-world deployment and scalability.

Validation axis	Metric	Target	Validation pathway	Benchmark/comparison logic
Swarm robotic platform	Average path deviation (APD)	<10%	Controlled testbed, simulation, and pilot missions in aquaculture and marina scenarios	Single robot vs. swarm operation; nominal vs. disturbed conditions
Digital twin fidelity	Normalised root mean square error (NRMSE)	<0.1	Comparison between DT estimates/predictions and *in-situ* measurements collected during pilot operations	DT-assisted estimation vs. direct measurements; static/manual interpretation vs. continuously updated DT
Human–swarm interaction trust	Trust perception scale for HRI (TPS-HRI)	>60%	User studies during supervised missions with non-roboticist stakeholders	DT/HSI-supported supervision vs. reduced supervisory support
Operator workload in human—swarm interaction	NASA task load index (NASA-TLX)	<50/100	User studies during supervised missions, including setup, alert, and reconfiguration	DT/HSI-supported supervision vs. reduced supervisory support
Operator interface usability	System usability scale (SUS)	>68%	End-user evaluation of the unity-based interface during representative monitoring/intervention tasks	Integrated REMORA UI vs. fragmented/manual operational workflow
Complementary operational indicators	Coverage efficiency, communication robustness, end-to-end latency, mission endurance, intervention frequency, alert response time, behaviour-reconfiguration time, supervised mission completion	Simulation, controlled tests, and field pilots in aquaculture and marina settings	Single robot vs. swarm; communications-aware vs. non-communications-aware behaviours; scenario-specific stress conditions	

Beyond these primary KPIs, REMORA will also report complementary operational indicators that are critical for deployment. These include coverage efficiency, communication robustness under multi-hop MANET conditions, end-to-end data latency, mission endurance, intervention frequency, alert response time, behaviour-reconfiguration time, and supervised mission completion. Importantly, these indicators will be analysed comparatively, for instance by contrasting single-robot and swarm deployments, communications-aware and non-communications-aware behaviours, or reduced vs. DT-assisted supervisory support. The two target pilots play distinct and complementary roles in this strategy: aquaculture provides a bounded but biologically dynamic environment to assess continuous environmental sensing, fish-related monitoring, and operator-assisted intervention, whereas the marina scenario stresses robustness, navigational safety, water-quality monitoring, and long-duration operation under more open and operationally heterogeneous conditions. In both cases, scalability will be assessed not only in terms of robot count, but also in terms of communication density, energy autonomy, maintenance burden, payload/calibration cost, and operator workload. Field robustness will be analysed against expected failure modes such as intermittent network partitions, loss or degradation of localisation, sensor drift, bio-fouling, actuator degradation, and partial robot dropout. In this way, the validation strategy is designed not only to quantify subsystem performance under nominal conditions, but also to assess whether the integrated architecture can degrade gracefully and sustain persistent, ecologically meaningful monitoring in realistic field conditions.

## 4 Conclusion

Consistent with the scope of a Perspective paper, this work does not claim mature field validation, but argues for a new systems-level paradigm for marine ecological monitoring. Marine ecosystems demand monitoring and intervention capabilities that exceed what single vehicles, sparse fixed sensors, or periodic manual sampling can realistically deliver at scale. We introduced REMORA as a 36-month initiative operationalising this shift through the integration of swarm intelligence, *in-situ* DTs, and HSI, with the goal of turning aquatic observability into a routine capability rather than an exceptional campaign.

Next steps focus on maturing the REMORA stack from early prototypes to sustained, in-the-wild operation: (i) scaling multi-robot behaviours that remain effective under intermittent connectivity and environmental disturbance; (ii) hardening the digital-twin pipeline for real-time assimilation, uncertainty-aware prediction, and “what-if” simulation loops; and (iii) designing and evaluating remote HSI mechanisms that let non-roboticist operators specify intent, manage exceptions, and calibrate autonomy without micromanagement.

## Data Availability

The original contributions presented in the study are included in the article/supplementary material, further inquiries can be directed to the corresponding author.
